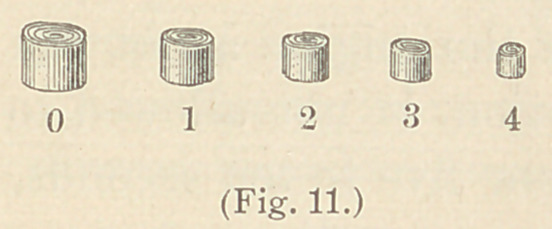# The Herbst Method of Filling Teeth

**Published:** 1887-02

**Authors:** C. F. W. Bödecker

**Affiliations:** New York


### THE HERBST METHOD OF FILLING TEETH.

---

BY C. F. W. BÖDECKER, D. D. S., M. D. S., NEW YORK.

---

Since the appearance of my last article on this subject, which was published in the INDEPENDENT PRACTITIONER for August, 1885, this system has been considerably modified, and to meet the numerous inquiries which are constantly coming to the writer, he has deemed it necessary very briefly to recapitulate its general principles. The advantages claimed for it are:

1st. Better adaptation to the walls of the cavity than it is possible to obtain by any other system.

2d. The saving of about one-half the time required for other methods.

3d. Some of the most difficult operations (as proximate surfaces of the molars and bicuspids) by this method are very easily performed.

4th. Gold can be perfectly adapted to the thin walls of enamel without danger of fracture.

5th. The introduction of gold, when done by this method, is much less annoying to the patient, and less laborious to the operator.

The instruments used for this method have been very much modified; they are mostly ordinary smooth burnishers, of which there are three sets; one set of engine points, one set of hand instruments,

(Fig. 1.)

and one set of bent hand instruments. The two former were designed by Herbst, the latter by Dr. Frank Abbott. Of the old set of steel engine instruments (see Fig. 1), only a few are now employed, although we may meet with cases in which all can be used. The most important of them is the roof-shaped instrument, No. 5, of which there should be several sizes. These can easily be made out of a broken bur, as follows: The broken instrument is put in the hand piece of the engine, which, while rotating rapidly, is ground upon an Arkansas stone, or sandpaper, No. 1. The instrument should lie obliquely upon the stone or sandpaper, like a pen in writing, and be quickly moved, drawing it from one side to the other.

The instruments Nos. 2, 3, and 4, are but seldom used for gold fillings. The larger instruments, Nos. 1, 2 and 6, are mostly intended for the use of amalgam and tin. The pointed instruments, Nos. 7 to 15, are used for finishing and condensing the edges of proximate fillings. To this set I have added three very small, round points, in shape resembling a round cavity bur. They are designed for the use of small proximate cavities in incisors, and to me have proved of great advantage. (See Fig. 2.)

(Fig. 2.)

Much more serviceable than steel rotating instruments are those made of agate, blood-stone or garnet. The advantages of stone over steel instruments is in the hardness of the former, which prevents the coherence of the gold to them. The former, therefore, may be run at a high rate of speed, and under considerable pressure, without perceptibly heating, and this permits the much more perfect condensation of the gold. Consequently, we observe that the surface of a gold filling, which has been condensed by means of an agate or garnet instrument, is very much harder than a filling which has been inserted by steel points. But a great drawback in the use of stone instruments is their liability to fracture. Herbst, while in this country, exhibited some agate points which had been set in such a manner that only about one thirty-second of an inch of the stone protruded from the steel mandrel, and they would withstand considerable pressure before breaking. These stone points, which in form somewhat resemble the steel instruments, Nos. 2, 3, 4, and 5, of fig. 1, are intended to be used for direct pressure, but for larger surfaces Herbst has of late employed round agate or garnet beads fastened upon a mandrel with sulphur, and these are intended to be used for lateral pressure, especially in finishing grinding or labial surfaces. Stone points should not be polished, but roughened upon a corundum stone, while rotating in the engine.

To secure a better union between the different layers of gold when stone instruments have been employed for condensing, the surface of the gold should be roughened by means of a serrated hand plugger, for which purpose Herbst employs a freshly broken excavator.

2 3 4 5 6

(Fig. 3.)

5 4 3 2

(Fig. 4)

The hand instruments designed by Herbst, (Fig. 4) are five in number. Four are pear-shaped and one is a very fine roof-shaped instrument. Nos. 1, 2, 3, 4 are intended to bring the gold to its proper place before the engine instruments are employed. No. 5 is an exploring instrument, which is to be pressed over the surface of the gold, especially the first layer, to discover the imperfectly condensed places.

Dr. Abbott's set (Fig. 3) is composed of bent burnishers, for those places which a straight instrument cannot perfectly reach.

One of the essential rules for filling by the Herbst method, is the conversion of all complicated cavities (such as proximate ones) which possess but one, two or three lateral walls, into simple ones (such as cavities involving the grinding surfaces of molars, and having four lateral walls), which is accomplished by the application of a proper matrix. The matrices used for this purpose are either made of steel, German silver, wood or shellac, or the Jack matrices may be employed. For the proximate surfaces of molars and bicuspids should be employed, if practical, either the German silver band matrix, the forms devised by Dr. Louis Jack, or a piece of watch-spring. The German silver band matrix can be very quickly made as follows: A piece of this metal, No. 32, about one inch in length and as wide as necessary, is bent around the tooth to be filled, in such a manner that the ends of the metal come to the buccal surface of the tooth. It is then firmly compressed around the tooth by means of a pair of pliers, made for that purpose. The ring is withdrawn from the tooth, a little soldering fluid (solution of chloride of zinc) applied, and over the point of an alcohol flame it is united by tin solder. If the tooth to be filled stands alone, the German silver matrix must be strengthened, either by soldering a thin brass wire around it, or by flowing tin solder upon the outside of the matrix wherever strength is required. In soldering, great care must be exercised that no tin runs to the inside of the matrix, especially that part which faces the cavity to be filled, for if, during the introduction of the gold, the tin is touched by the rotating instrument, some of it will be incorporated into the filling and impair the cohesion of the separate layers of gold. It is also of great importance to clean the matrix thoroughly after soldering, which can best be done with the dental engine, by means of a piece of cotton wound around an old engine bur dipped in moistened pumice stone. When this form of matrix is employed, it is advisable to prepare it previous to excavating the cavity, for the contour of the tooth can thus be preserved much more easily.

The depressed matrices devised by Dr. Louis Jack are sufficiently well known to require no further description. The watch-spring matrices are made out of a piece of watch-spring saw, such as may be obtained from any of the dental depots, in the following manner: A piece of saw, about half an inch long and as broad as the cavity is deep, is cut off and heated over a spirit flame until it is dark blue. The points of the matrix which are designed to rest on the cervical edge of the cavity should be well rounded off, that in cavities extending under the gum it may be pushed down without injuring either the lingual or buccal portion of the gum. The lateral ends of the matrix must be bent around the lingual and buccal portion of the tooth to be filled, like a clasp. When thus prepared it may be secured by one or two wedges of wood, or ordinary pins, inserted, one from the buccal the other from the lingual side. These wedges should be placed near the gum, between the matrix and the adjoining tooth, firmly pressing the former against the edges of the cavity. In adjusting a matrix care should be observed that in all mesial cavities it does not quite reach the grinding surface of the tooth, or it will obstruct the entrance to the cavity. All the steel matrices may be saved and used many successive times. When two cavities in bicuspids or molars face each other, if the former plan does not answer, the matrix, after it has been placed in position, may be secured by filling one of the cavities with cotton or shellac.

For filling cavities in the proximate surfaces of the teeth, when they are opened from either the labial, the buccal, or the lingual side, we may employ, as a matrix, a piece of thin steel spring about four to six inches long, and one-eighth to one-fourth of an inch wide. Across one of the ends of this spring a piece of German silver or brass tubing may be fastened with tin solder, in such a manner that, when the spring is in position between the teeth to be filled, the tubing will prevent it from being pulled through. In other instances, one end of the steel spring may be fastened into a small piece of shellac, and while this is yet in a soft condition the whole may be pressod into the desired position. This form of matrix is especially applicable for teeth with large crowns and narrow necks, such as lower bicuspids, when the steel spring without the shellac would impinge upon the gum. This steel spring may also be used as a protection to a neighboring tooth during the preparation of the cavity.

In some instances, where the lingual walls of upper incisors, to be filled from the labial surface, are not broken away, we may employ as a matrix a piece of German silver about one inch in length, and wide enough to completely cover the cavity in the lingual surface of the tooth to be filled. Insert it between the proximate surfaces of the incisors containing the cavity, and bend one end of it so as to cover the cavity in the lingual surface; the other end is bent out of the way, over the labial surface of the adjoining tooth. (See Figure 5.)

(Fig. 5.)

If, on the other hand, we intend to fill a cavity from the lingual surface of an incisor tooth, the matrix must be reversed. (See Fig. 6.)

(Fig. 6.)

For filling the proximate surfaces of incisors when their lingual walls are much broken, as well as in contour operations, a matrix of shellac is employed, which may be made in the following manner: A piece of shellac, the size of a large walnut, is warmed over an alcohol lamp to the consistency of putty, and after the rubber dam has been adjusted, this is pressed against the lingual wall, extending a little over the cutting edges of four or six of the teeth. After it has become hard it is again removed from the mouth, cooled in water, and a small piece of steel spring warmed over the flame of an alcohol lamp is inserted in the shellac at the place corresponding to the proximate surfaces of the tooth or teeth to be filled, and, while yet warm, the matrix is replaced in the mouth and adjusted as required. The piece of steel spring must not quite reach the labial surface of the tooth, as it may offer an obstruction to the entrance of the cavity during the introduction of the gold. (See Figs. 7 and 8.)

(Fig. 7.)

Representing a shellac matrix with two pieces of steel spring (*b*) inserted.

(Fig. 8.)

The same matrix when in situation. *b b* steel springs.

The matrices used for contour operations of incisors are made in a similar manner, but besides the steel matrix of the proximate surfaces, an additional one should be inserted corresponding to the cutting edge of the tooth to be restored. (See Figs. 9 and 10 *b. b.*)

(Fig. 9.)

Shellac matrix for contour operations.

During the introduction of the filling material, the gold, which (when unannealed) apparently shows no signs of cohesion, working as soft as tin foil, when burnished, becomes somewhat cohesive. A satisfactory explanation of this fact has as yet not been given by any one, and although this property will be observed in the gold from every manufacturer, Wolrab's German gold possesses it in a very marked degree.

(Fig. 10.)

The matrix in position after the introduction of the gold. *a*, shellac; *b*, steel springs adjusted against the cutting and proximate surfaces of central; *c*, gold filling.

The forms of gold best adapted for this method of filling are very soft cylinders (see Fig. 11), especially in the beginning of the operation; in the middle and upon the surface of a filling any preparation may be employed, although for large surfaces heavy foil (Nos. 30 to 60) applied in narrow strips gives the best results. If foil is used for the first layers of the operation, Nos. 3, 4 and 5 are the best for the purpose. The leaves are cut into halves, and rolled into a rope between the fingers, or with a napkin, and cut into pellets of required length; or the sheet may be divided into squares measuring from one-half to one inch, which, by means of a pair of foil tweezers or the fingers are formed into pellets. The foil, as well as the cylinders, should never be annealed when used in the first layers of the cavity, except it be a contour operation.

0 1 2 3 4

(Fig. 11.)

The main rule to be observed in the starting of a filling is, that the first layer must be sufficiently large, so that when condensed it will lie securely in the cavity without being supported by an instrument. When too little gold has been put into the first layer, or when a number of too small cylinders are used and an attempt is made to condense them, the gold will roll about under the instrument and become too hard to be again adapted to the walls and edges of the cavity. The same condition will be observed when the first hand instrument used in condensing the gold has been too small. In very large and flat cavities containing but little undercut, the first layer of gold may be condensed by means of cotton, as follows: For a large cavity, introduce from five to eight large, soft gold cylinders, without attempting to condense them. A piece of chemically pure cotton, as large as the cavity will hold, is then inserted in the cavity, and, by means of a rotating burnisher in the engine, it is pressed into every part of the cavity. After the cotton is removed, the gold is further condensed into every depression with agate points.

The Herbst hand instruments (Fig. 4), while pressing hard upon the gold, are rotated in the hand about one-half or three-quarters of a turn, but the Abbott instruments are merely moved from side to side. By a rotary motion the gold is much better condensed than by simple pressure. Before the hand instruments are used, they should be rubbed upon a piece of No. 1 sandpaper. After the gold has been thus condensed, the perfect adaptation is obtained by a roof-shaped point, made of steel or agate, in the engine. After the instrument is passed over a piece of sandpaper and is perfectly clean, it is, while rotating, pressed firmly upon the gold, condensing it thoroughly into every depression of the cavity. In condensing, this instrument should not be held upon one spot, but be moved around, and especially along the edges of the cavity. In using steel points, care should be taken that the engine is not run too fast, and that the burnisher, while in motion, is not allowed to be in contact with the gold longer than from five to ten seconds, lest the gold be heated to such an extent as to cause discomfort, or even great pain to the patient. When the first layer of gold has been thoroughly condensed with the roof-shaped instruments, the hand instrument No. 5 (Fig. 4), while rotating, is pressed firmly around the edges and depressions of the cavity. If this makes any deep pits in the gold, it proves that in these places it was not perfectly condensed, and a smaller roof-shaped instrument than that used in the first instance should be employed in the engine to condense these places. All deep pits present in the layer of gold should now be filled up with very small gold cylinders, and thoroughly condensed until the surface of the gold is even. If stone instruments have been employed for condensing, the gold should be roughened by a serrated hand plugger (a freshly broken excavator), or a rotating steel point in the engine, before another layer of gold is added. All the succeeding layers of gold are manipulated in the same manner, except upon larger surfaces, where we can employ the garnet or agate bead with lateral pressure, when heavy foil (Nos. 30 to 60) will be found to give better results. This may be packed upon the other layer of gold in single strips, burnishing every piece down by means of the rotating instrument, while directing the foil by means of a pair of tweezers in the same manner as in packing heavy foil by the electro-magnetic or mechanical mallet. In these instances, when the garnet or agate bead has been made rough, we need not use the hand instrument to roughen each layer of gold. Herbst, however, introduces a rather thick layer of heavy foil first, and then uses the agate or garnet bead; but in these instances considerable pressure is required to condense the gold perfectly. In some situations, as in buccal walls of molars and bicuspids, when the gold cannot be condensed by direct action of the instrument, the right angle attachment, or an Abbott hand instrument should be employed.

As this method of filling teeth—like every other—requires some practice, the writer deems it safer for a beginner to finish the operation in the old accustomed manner.

Tin is introduced in the same manner as gold, either in the form of foil or as Robinson's metal. Nos. 4 to 6 foil is cut in half, and is made into a rope with the fingers or a napkin, and cut into pieces of the desired length, which ought to be used when prepared.

Cavities in front teeth, which it is intended to fill with amalgam or oxy-phosphate, may be lined with a thin layer of gold, which will impart to the thin wall of enamel a very life-like appearance. The method is as follows: A large and very soft gold cylinder is compressed between the fingers and immersed in a thin solution of gum-copal (about 2 grs. of gum-copal to ½ oz. of sulphuric ether), which is used for the purpose of preventing the mercury of the amalgam from uniting with the gold, and thus discoloring the tooth. The surplus liquid is pressed out with the fingers, the ether allowed to evaporate, and then, by means of a piece of cotton, the gold is pressed into the cavity and thoroughly condensed by a rotation instrument in the engine pressed firmly upon the cotton. Upon the removal of the cotton it will be found that the thin layer of gold has been thoroughly and uniformly adapted to every part of the cavity, which may then be filled, either with amalgam or cement, without future discoloration.

## Figures and Tables

**Fig. 1. f1:**
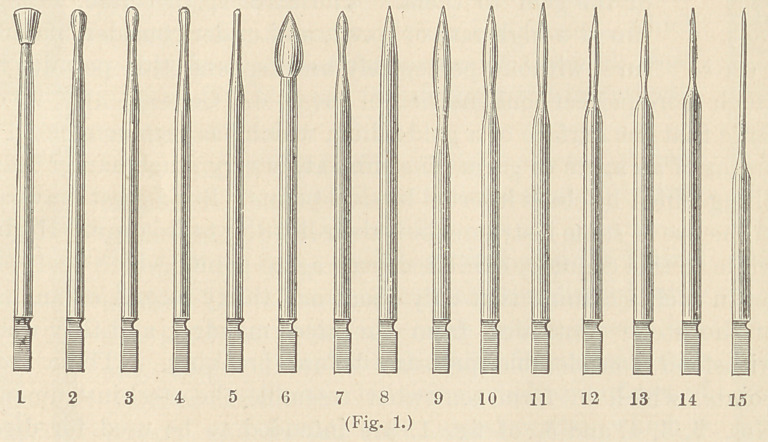


**Fig. 2. f2:**
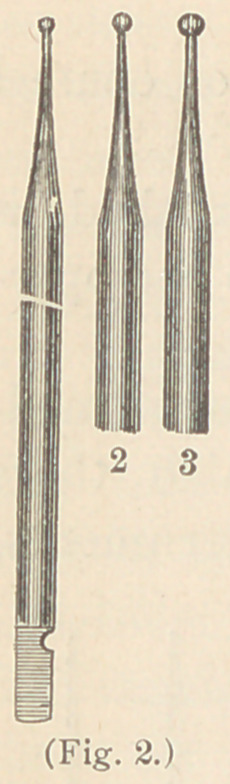


**Fig. 3. f3:**
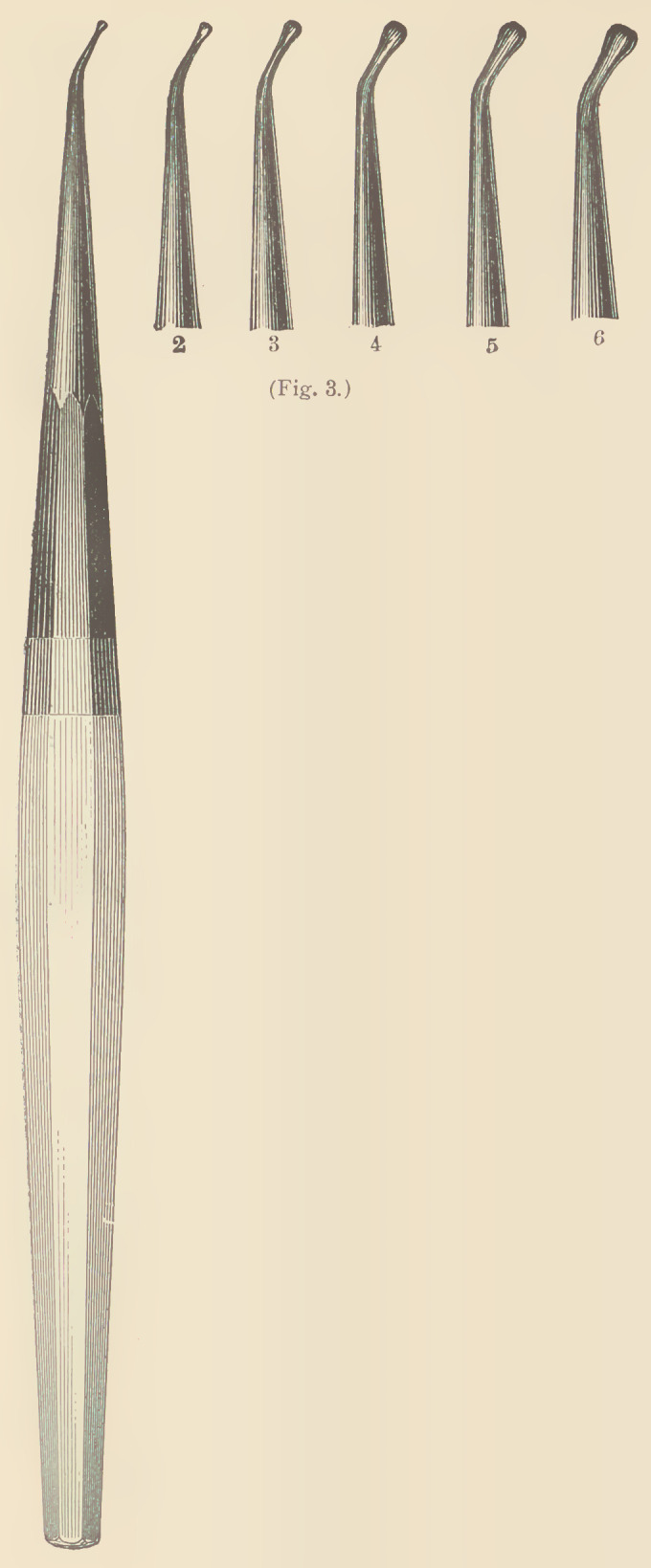


**Fig. 4. f4:**
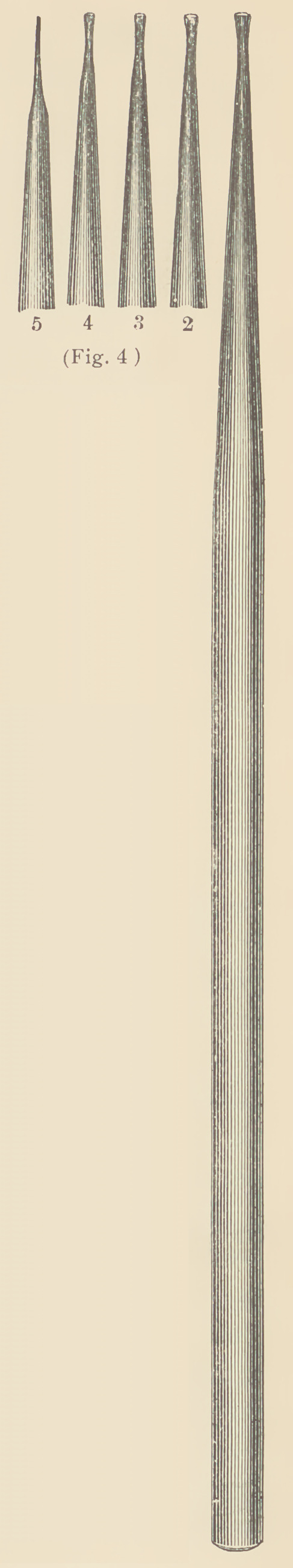


**Fig. 5. f5:**
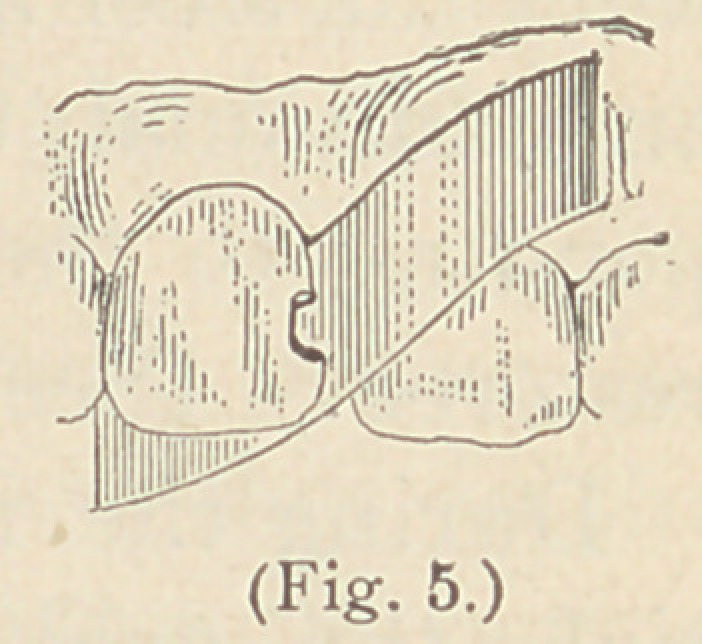


**Fig. 6. f6:**
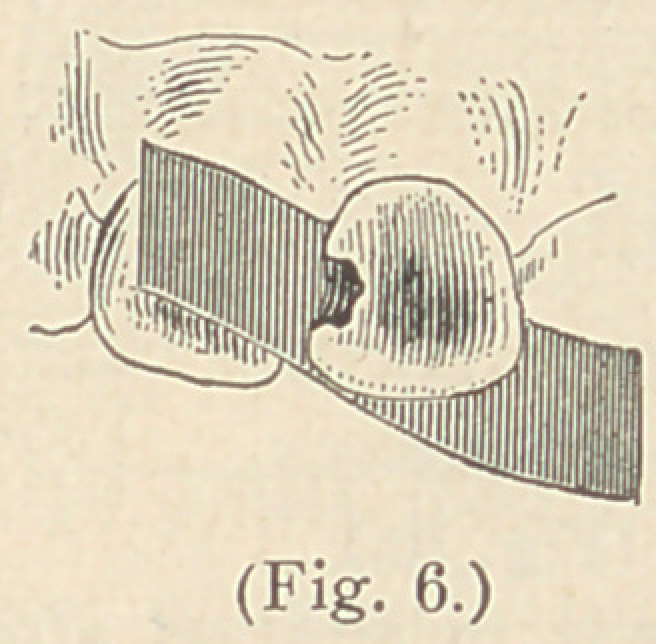


**Fig. 7. f7:**
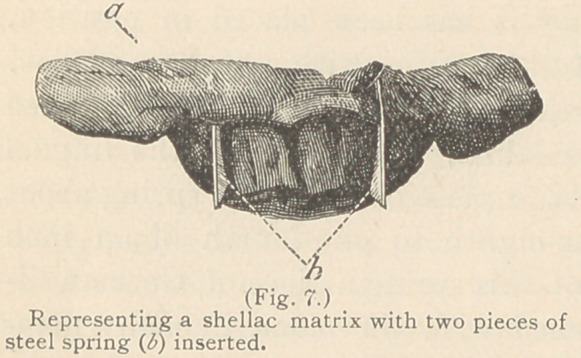


**Fig. 8. f8:**
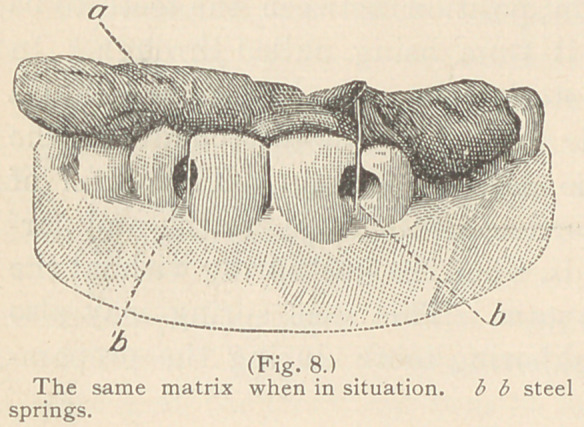


**Fig. 9. f9:**
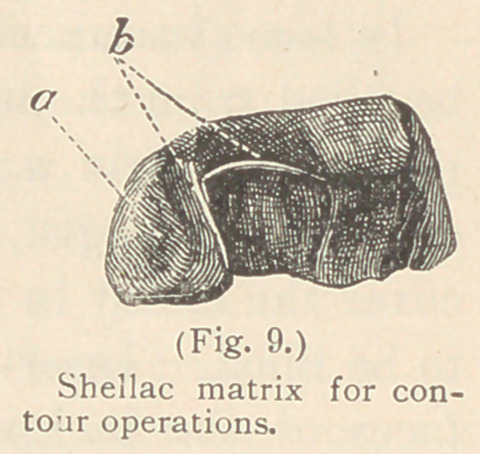


**Fig. 10. f10:**
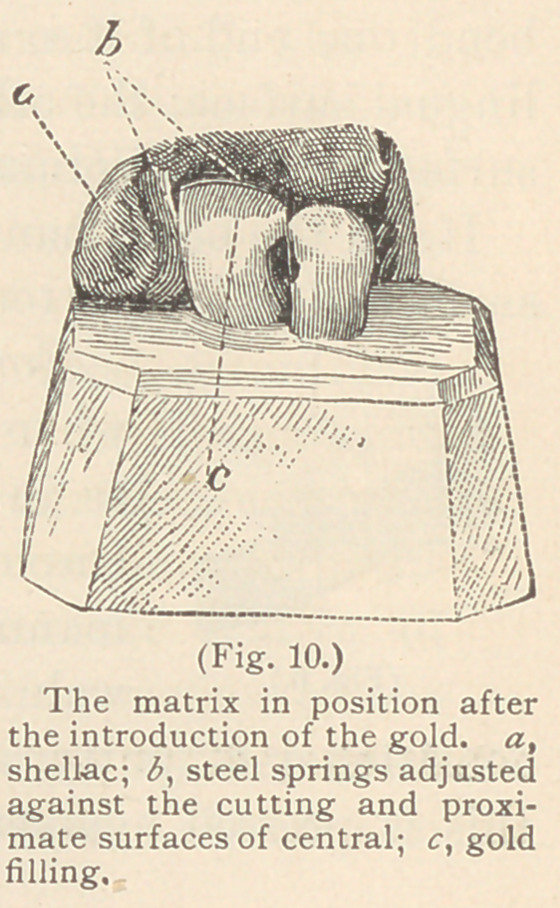


**Fig. 11. f11:**